# Savolitinib versus crizotinib for treating MET positive non‐small cell lung cancer

**DOI:** 10.1111/1759-7714.14848

**Published:** 2023-03-21

**Authors:** Kang Miao, Xiaotong Zhang, Hanping Wang, Xiaoyan Si, Li Zhang

**Affiliations:** ^1^ Department of Pulmonary and Critical Care Medicine, Peking Union Medical College Hospital Chinese Academy of Medical Sciences & Peking Union Medical College Beijing China

**Keywords:** crizotinib, MET amplication, METex14 skipping, savolitinib

## Abstract

**Background:**

The c‐MET protein, encoded by the mesenchymal‐epithelial transition factor (MET) gene, can regulate cell proliferation, migration and invasion. Studies have shown that it is one of the essential driver genes for non‐small cell lung cancer (NSCLC). Currently, several clinical studies have carried out objective assessments on the efficacy and safety of different types of MET tyrosine kinase inhibitors (TKIs). However, direct cross‐sectional comparisons between different agents are still not available.

**Methods:**

Our study was a single‐center retrospective clinical study, which collected the data from MET positive NSCLC patients treated with MET TKIs at the Lung Cancer Center of Peking Union Medical College Hospital. We explored the efficacy and safety of crizotinib versus savolitinib in patients with METex14 skipping and MET amplification, separately.

**Results:**

Patients with METex14 skipping (median PFS = 10.7 months) had a better clinical response to MET TKIs than MET amplification patients (median PFS = 4.1 months). In the METex14 skipping subgroup, savolitinib did not show better survival benefit with significance than crizotinib (*p* > 0.05). In the MET amplification subgroup, savolitinib (median PFS = 7.1 months) demonstrated a better progression‐free survival benefit than crizotinib (median PFS = 1.4 months), *p* = 0.05. The most common adverse effects of both MET TKIs were peripheral edema (41.2%), gastrointestinal reactions (23.5%), and liver injury (14.7%). The incidence rate of peripheral edema was higher in savolitinib than crizotinib.

**Conclusion:**

In METex14 skipping NSCLC patients, the efficacy of savolitinib and crizotinib did not show significant difference. In MET amplification patients, savolitinib showed better efficacy than crizotinib.

## Graphical abstract

Our study was a single‐center retrospective clinical study, which collected the data from MET positive NSCLC patients treated with MET TKIs at the Lung Cancer Center of Peking Union Medical College Hospital. In METex14 skipping subgroup, savolitinib did not show better survival benefit with significance than crizotinib (*p* > 0.05). In MET amplification subgroup, savolitinib (median PFS = 7.1 months) demonstrated a better progression‐free survival benefit than crizotinib (median PFS = 1.4 months), *p* = 0.05.

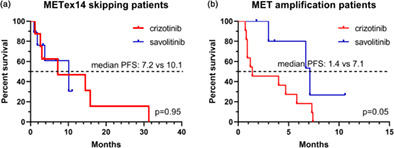

## INTRODUCTION

Lung cancer is one of the leading causes of mortality, of which non‐small cell lung cancer (NSCLC) accounts for approximately 85%.^1^ In recent years, the progress in targeted therapy has brought significant survival benefits to patients with advanced NSCLC. For example, gefitinib, afatinib, and oxitinib for epidermal growth factor receptor (EGFR) mutations, crizotinib, alectinib, and lorlatinib for anaplastic lymphoma kinase (ALK) fusions.^2^ In addition, there are some targets that are rarely detected but are similarly important tumor driver genes. For instance, kirsten rat sarcoma viral oncogene homolog (KRAS) mutations, recombinant c‐ros oncogene 1 (ROS1) rearrangements, neurotrophin receptor kinase (NTRK) fusions, rearranged during transfection (RET) rearrangements, human epidermal growth factor receptor‐2 (HER2) mutations, and mesenchymal‐epithelial transition factor (MET) alterations.^3^

The MET gene is located in the chromosome 7q21‐31 and encodes c‐MET protein, a tyrosine kinase receptor for hepatocyte growth factor. It is capable of regulating cell proliferation, migration and invasion by activating the downstream MEK–ERK pathway and PI3K pathway.^4^ The oncogenic driving variants of MET gene mainly include METex14 skipping and MET amplification.^5^ METex14 encodes a structural domain containing the ubiquitin ligase binding site of Y1003.^6^ METex14 skipping is present in about 1%–3% of NSCLC patients and may cause deletion of the binding sites of Y1003 and c‐CBL, abnormal degradation of MET proteins and sustained activation of MET gene.^6^ MET amplification can exist either independently as a primary driver gene as well as in combination as a secondary gene following resistance to EGFR tyrosine kinase inhibitors (TKIs) or other targeted therapy drugs.^7^

MET TKI can be classified into type Ia, type Ib, type II and type III according to its mechanism.^8^ Crizotinib, an ATP‐competitive MET/ALK/ROS1 multitarget inhibitor, is a type Ia MET TKI that inhibits the phosphorylation of the receptor by blocking ATP conjugation.^9^ Savolitinib is a type Ib MET TKI that also binds ATP but does not interact with G1163. It has a higher specificity than crizotinib.^10^ A type II MET TKI (e.g., cabozantinib) competitively inhibits the hydrophobic band adjacent to the ATP binding site, while type III MET TKIs inhibit the metastable state.^11^ Currently, multiple prospective clinical studies provide an objective assessment of the efficacy and safety of MET TKIs. However, direct cross‐sectional comparisons between different types of MET TKIs are still lacking.

Herein, we conducted a single‐center retrospective observational study to explore the efficacy and safety of two types of MET TKIs available in China (crizotinib vs. savolitinib) in METex14 skipping and MET amplification NSCLC patients. By analyzing data from real‐world applications, we hope to provide a basis for further optimization of clinical decisions in selecting MET TKIs.

## METHODS

### Study objective

This was a single‐center retrospective clinical study to explore the difference in efficacy and safety of crizotinib versus savolitinib in NSCLC patients with METex14 skipping and MET amplification. The study design was in accordance with the ethical guidelines of the Declaration of Helsinki and approved by the ethical review committee of Peking Union Medical College Hospital.

### Inclusion criteria

In this study, we collected and analyzed data from MET positive NSCLC patients treated with crizotinib or savolitinib at Peking Union Medical College Hospital from January 2018 to June 2022. Inclusion criteria was as follows: (1) Diagnosis of NSCLC by histology or cytology; (2) tumor stage IVa or IVb; (3) at least one measurable tumor lesion; (4) presence of METex14 skipping or MET amplification (gene copy number ≥2) identified by next‐generation sequencing (NGS) or fluorescence in situ hybridization (FISH) assay (NGS: A genetic sequencing technology that extracts DNA from the tumor tissues of patients and detects the presence of mutations in various tumor driver genes, including MET; FISH: A genetic diagnostic technique that utilizes fluorescent moieties to label DNA probes, which then hybridize in situ to sample DNA and are counted under the fluorescent microscope) and (5) received crizotinib or savolitinib treatment and underwent at least one‐time radiographic evaluation.

### Data collection

For each patient, we obtained the following information separately: gender, age, pathology type, disease stage, smoking history, eastern cooperative oncology group (ECOG) score, *EGFR* mutation status, MET mutation status (additional gene copy number was recorded for MET amplification patients), MET TKI type, treatment lines for MET TKI, baseline tumor dimensions, optimum tumor size change, progression‐free survival, and drug‐related adverse reactions.

### Data analysis

IBM SPSS version 24.0 was used to complete statistical analyses, and graph pad prism version 8.0.1 was used to draw statistical graphs. We analyzed the characteristics of basic information of patients and their treatment strategy with MET TKIs. The patients were grouped according to their MET mutation type. The efficacy of MET TKI was reflected by objective response rate (ORR) and progression‐free survival (PFS), while safety was demonstrated by the incidence of adverse effects. Progression‐free survival was defined as the time from initiation of MET TKI to imaging assessment of lesion progression >20%. Count data was described according to its statistical quantity and percentage, while measurement data was converted into count data by self‐defined stratification. In METex14 skipping and MET amplification subgroups, basic information was further matched according to MET TKI type (crizotinib vs. savolitinib). Correlation between different count data was verified by chi‐square test, while correlation between measurement data was verified by linear regression, and *R* ^2^ reflected the fitting degree of the model. A waterfall map was drawn to visually reflect the maximum change value of tumor size in all patients. The survival benefit among different groups was reflected by Kaplan Meier curve. A log‐rank test was used to explore the influence of different MET TKI and other interfering factors on MET positive patients. The hazard ratio (HR) and *p*‐value from Cox regression were reported. *p*‐values <0.05 were considered statistically significant.

## RESULTS

We collected the information of 56 NSCLC patients who were MET positive in the lung cancer center of Peking Union Medical College Hospital from January 2018 to June 2022. Among them, 34 patients who had been treated with MET TKIs and experienced at least one radiographic evaluation, were formally included in our study (Table S1).

Of these 34 patients, 21 (61.8%) were male and 13 (38.2%) were female, with no significant differences between METex14 skipping and MET amplification patients. The median age was 62 years old. The METex14 skipping population was dominated by patients over 60 years old (82.4%), whereas more than half of the MET amplification population was made up of young patients (less than 60 years old). The pathological type was mainly adenocarcinoma (94.1%), with only two patients of other types. All patients included in our study had stage IV NSCLC, of which 41.2% were at stage IVa and 58.8% were at stage IVb. Nearly half of the patients had a smoking history (47.1%). At the time of MET TKI initiation, 76.5% of patients were in good general condition (ECOG score 0–1). MET amplification is one of the essential mechanisms of EGFR TKI resistance. All MET amplification patients included in this study had *EGFR* mutations, while only three METex14 skipping patients had *EGFR* mutations. A MET TKI was more commonly used as the first‐line treatment (58.8%) for METex14 skipping due to its independent oncogenic characteristic. In contrast, it was used more frequently for post‐line therapy (88.2%) for MET amplification. In terms of MET TKI selection, 55.9% of patients selected crizotinib while 44.1% chose savolitinib (Table 1).

**TABLE 1:** Basic information for METex14 skipping and MET amplification patients.

Total	Patient		METex14 skipping		MET amplification		*p*‐value
	34	100.0%	17	100.0%	17	100.0%	
Sex							0.724
Male	21	61.8%	11	64.7%	10	58.8%	
Female	13	38.2%	6	35.3%	7	41.2%	
Age	62 (57–70)						0.073
<60	12	35.3%	3	17.6%	9	52.9%	
≥60	22	64.7%	14	82.4%	8	47.1%	
Histology							0.466
Adenocarcinoma	32	94.1%	15	88.2%	17	100.0%	
Other	2	5.9%	2	11.8%	0	0.0%	
Stage							0.727
IVa	14	41.2%	8	47.1%	6	35.3%	
IVb	20	58.8%	9	52.9%	11	64.7%	
Smoking status							0.303
No	18	52.9%	7	41.2%	11	64.7%	
Yes	16	47.1%	10	58.8%	6	35.3%	
ECOG PS							1.000
0	10	29.4%	5	29.4%	5	29.4%	
1	16	47.1%	8	47.1%	8	47.1%	
2–4	8	23.5%	4	23.5%	4	23.5%	
*EGFR* mutation							0.001
No	17	50.0%	14	82.4%	0	0.0%	
EGFR‐19 del	9	26.5%	3	17.6%	6	35.3%	
EGFR‐21 L858R	8	23.5%	0	0.0%	11	64.7%	
Line of therapy							
First‐line	12	35.3%	10	58.8%	2	11.8%	0.015
Second‐line	11	32.4%	4	23.5%	7	41.2%	
Third‐line and beyond	11	32.4%	3	17.6%	8	47.1%	
MET‐TKI type							0.490
Crizotinib	19	55.9%	8	47.1%	11	64.7%	
Savolitinib	15	44.1%	9	52.9%	6	35.3%	

Abbreviation: MET, mesenchymal‐epithelial transition factor; TKI, tyrosine kinase inhibitor.

**FIGURE 1:**
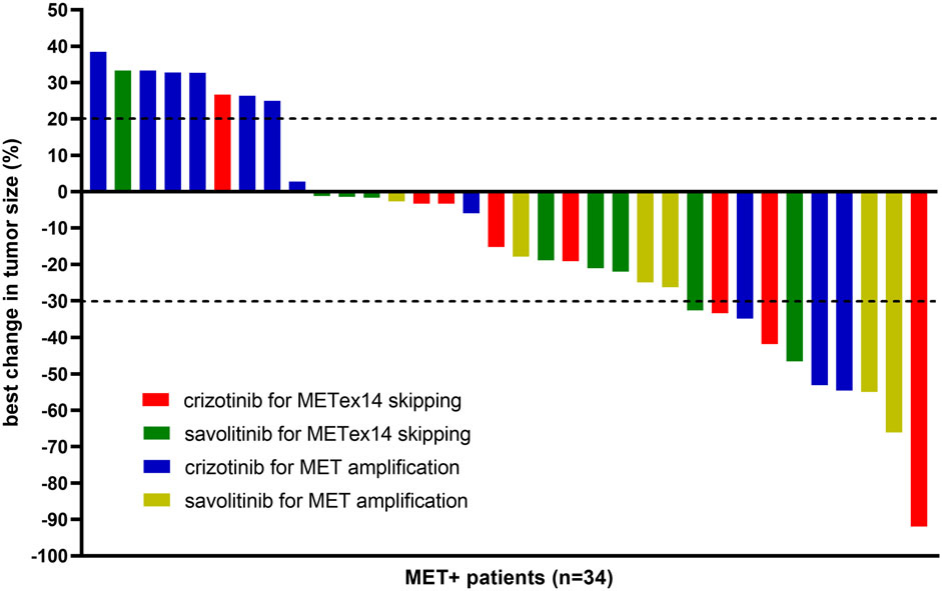
Best change in tumor size.

**FIGURE 2:**
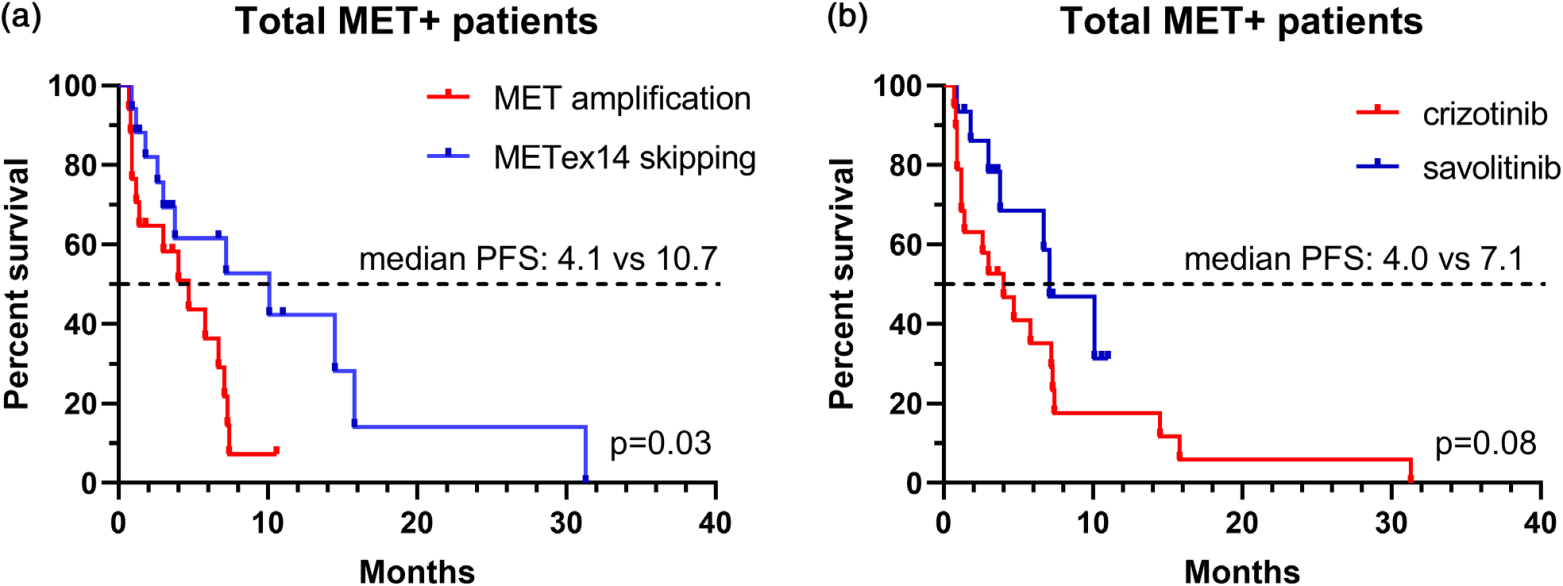
Progression‐free survival curve. (a) Mesenchymal‐epithelial transition factor (MET) amplification versus METex14 skipping. (b) Crizotinib versus savotinib.

The maximum change in tumor diameter of all patients is shown in Figure 1. In the METex14 skipping subgroup, five (29.4%) patients achieved partial response (PR), 10 (58.8%) patients had stable disease (SD), and two (11.8%) patients had progressive disease (PD). In contrast, in the MET amplification subgroup, five (29.4%) patients achieved PR, six (35.3%) had SD, and six (35.3%) had PD. There was a total of eight patients who completely failed to respond to MET TKIs, six of whom were in the crizotinib‐treated MET amplification subgroup. MET TKIs were significantly more effective for METex14 skipping patients (median PFS: 10.7 months, 95% confidence intervals: 5.2–17.3 months) than MET amplification patients (median PFS: 4.1 months, 95% confidence intervals: 3.0–5.9 months) (Figure 2a). In addition, there were discrepancies in the efficacy of different MET TKI, with savolitinib (median PFS: 7.1 months, 95% confidence intervals: 5.2–9.3 months) providing a greater survival benefit than crizotinib (median PFS: 4.0 months, 95% confidence intervals: 1.5–6.9 months) (Figure 2b).

**FIGURE 3:**
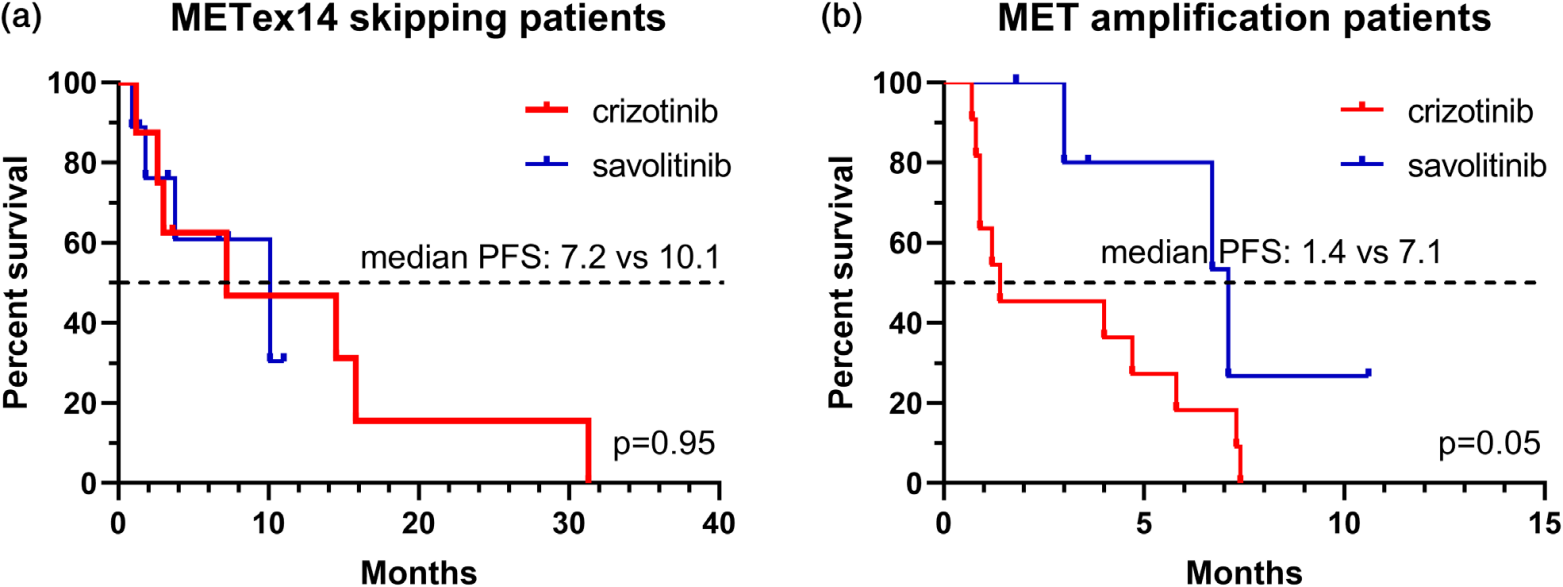
Subgroup analysis of progression‐free survival curve. (a) METex14 skipping subgroup. (b) Mesenchymal‐epithelial transition factor (MET) amplification subgroup.

In the METex14 skipping subgroup, savolitinib (median PFS: 10.1 months, 95% confidence intervals: 4.4–19.6 months) showed numerically longer median PFS than crizotinib (median PFS: 7.2 months, 95% confidence intervals: 3.6–19.5 months), with no statistical difference (*p* = 0.95) (Figure 3a). Further, we performed a matched analysis for other potential interfering variables. The results showed that none of the gender, age, disease stage, smoking history, ECOG score, *EGFR* mutation status, or treatment lines showed statistical differences between crizotinib and savolitinib (Table 2).

**TABLE 2:** Basic information between crizotinib and savolitinib for METex14 skipping.

MET‐TKI	Crizotinib (*N* = 8)		Savolitinib (*N* = 9)		*p*‐value
Sex					0.492
Male	4	50.0%	7	77.8%	
Female	4	50.0%	2	22.2%	
Age					0.910
<60	2	25.0%	1	11.1%	
≥60	6	75.0%	8	88.9%	
Stage					0.819
IVa	4	50.0%	4	44.4%	
IVb	4	50.0%	5	55.6%	
Smoking status					0.839
No	4	50.0%	3	33.3%	
Yes	4	50.0%	6	66.7%	
ECOG PS					0.552
0	3	37.5%	2	22.2%	
1	4	50.0%	4	44.4%	
2–4	1	12.5%	3	33.3%	
*EGFR* mutation					0.453
No	6	75.0%	8	88.9%	
EGFR‐19 del	2	25.0%	1	11.1%	
EGFR‐21 L858R	0	0.0%	0	0.0%	
Line of therapy					0.432
First‐line	6	75.0%	4	44.4%	
Second‐line	1	12.5%	3	33.3%	
Third‐line and beyond	1	12.5%	2	22.2%	

Abbreviation: MET, mesenchymal‐epithelial transition factor; TKI, tyrosine kinase inhibitor.

**FIGURE 4:**
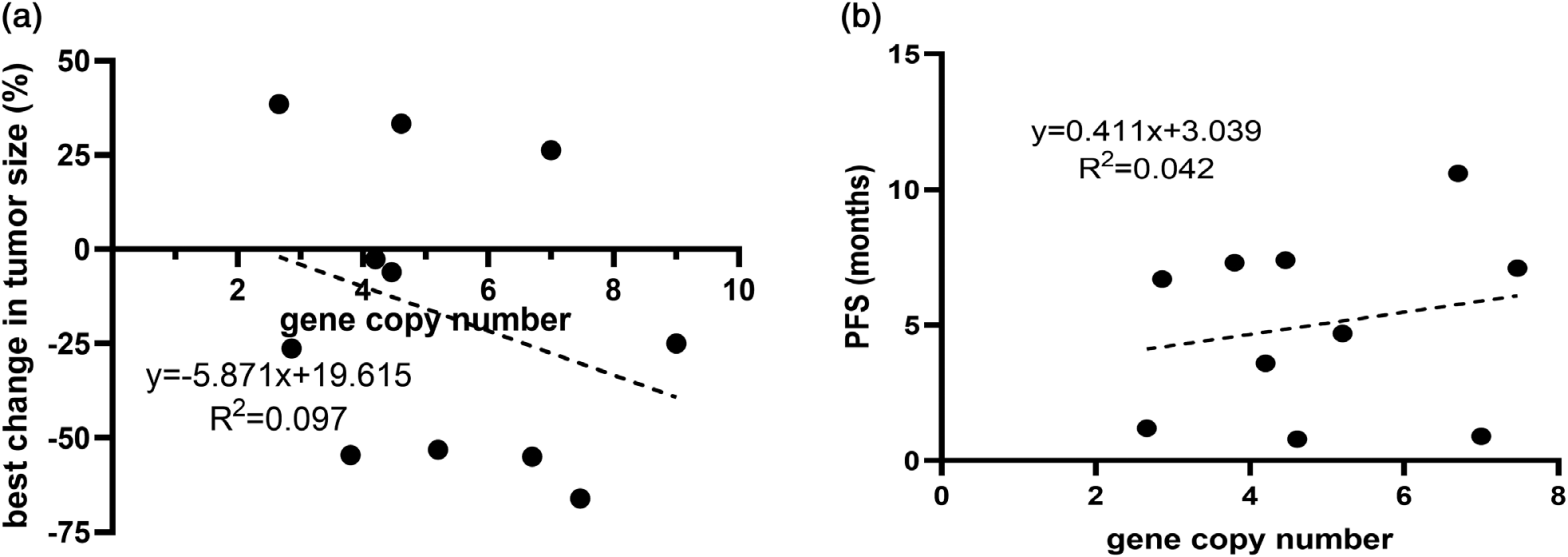
Correlation analysis between gene copy number and prognosis for mesenchymal‐epithelial transition factor (MET) amplification. (a) Correlation of gene copy number with best change in tumor size. (b) Correlation of gene copy number with progression‐free survival.

In the MET amplification subgroup, savolitinib (median PFS: 7.1 months, 95% confidence intervals: 4.7–9.5 months) demonstrated better survival benefit than crizotinib (median PFS: 1.4 months, 95% confidence intervals: 0.1–4.8 months), *p* = 0.05 (Figure 3b). Matching analysis likewise indicated that the basic information of patients was not found to be notably different in the two MET TKI subgroups (Table 3). Further, we performed a Cox regression analysis of the variables in the MET amplification subgroup. The results showed that the type of MET TKI was the greatest prognostic impact, with savolitinib showed trend of better benefit than crizotinib, HR = 0.331, *p* = 0.042 (Table 4). Also, we explored the effect of gene copy number (GCN) on prognosis in this subgroup. The results showed that the tumors tended to shrink and PFS was extended in response to the increase of GCN (Figure 4). However, the predictive efficacy of GCN was not satisfactory for either the degree of tumor shrinkage (*R* ^2^ = 0.097) or extension time of PFS (*R* ^2^ = 0.042).

**TABLE 3:** Basic information between crizotinib and savolitinib for MET amplification.

	Crizotinib (*N* = 11)		Savolitinib (*N* = 6)		*p*‐value
Sex					0.976
Male	7	63.6%	3	50.0%	
Female	4	36.4%	3	50.0%	
Age					0.742
<60	5	45.5%	4	66.7%	
≥60	6	54.5%	2	33.3%	
Stage					0.512
IVa	5	45.5%	1	16.7%	
IVb	6	54.5%	5	83.3%	
Smoking status					0.901
No	7	63.6%	4	66.7%	
Yes	4	36.4%	2	33.3%	
ECOG PS					0.224
0	3	27.3%	2	33.3%	
1	4	36.4%	4	66.7%	
2–4	4	36.4%	0	0.0%	
*EGFR* mutation					0.885
No	0	0.0%	0	0.0%	
EGFR‐19 del	4	36.4%	2	33.3%	
EGFR‐21 L858R	7	63.6%	4	66.7%	
Line of therapy					0.370
First‐line	2	18.2%	0	0.0%	
Second‐line	5	45.5%	2	33.3%	
Third‐line and beyond	4	36.4%	4	66.7%	

Abbreviation: MET, mesenchymal‐epithelial transition factor.

**TABLE 4:** Univariate Cox regression (MET amplification).

HR	*p*‐value	
MET‐TKI type		
Crizotinib	Reference	
Savolitinib	0.331	0.042
Sex		
Male	Reference	
Female	0.675	0.484
Age		
<60	Reference	
≥60	0.489	0.234
Stage		
IVa	Reference	
IVb	2.291	0.254
Smoking status		
No	Reference	
Yes	1.054	0.925
ECOG PS		
0	Reference	
1	1.071	0.360
2–4	2.147	0.380
*EGFR* mutation		
No	Reference	
EGFR‐19 del	0.981	0.997
EGFR‐21 L858R	0.955	0.955
Line of therapy		
First‐line	Reference	
Second‐line	1.118	0.896
Third‐line and beyond	1.300	0.761

Abbreviation: MET, mesenchymal‐epithelial transition factor; TKI, tyrosine kinase inhibitor.

For the purpose of assessing the safety of medication administration, we estimated the incidence of the three most common adverse reactions (peripheral edema, gastrointestinal reactions, and liver injury). Approximately, 64.7% patients using a MET TKI experienced these drug‐related adverse reactions; the incidence of peripheral edema was 41.2%, gastrointestinal reactions was 23.5% and liver injury was 14.7%. Adverse reactions occurred in 52.6% patients in the crizotinib group, with an incidence of 26.3% for edema, 15.8% for gastrointestinal reactions, and 15.8% for liver injury. Grade 3 or higher adverse reactions occurred in 10.5% patients, mainly consisting of liver injury, while in the savolitinib group, 73.3% of patients experienced MET TKI‐related adverse reactions, with 60.0% edema, 26.7% gastrointestinal reactions and 13.3% liver injury. Among them, the incidence of grade 3 or higher adverse reactions was 26.7%, including two cases of liver injury, one edema and one edema combined with gastrointestinal reaction.

## DISCUSSION

With an increasing number of MET TKIs approved for marketing, selecting a suitable agent for patients has become an urgent need for clinicians. Currently, the approved MET TKIs include crizotinib, savolitinib, capmatinib, and tepotinib.^12^ From the perspective of accessibility, the available MET TKIs in China cover crizotinib and savolitinib. Hence, our study cross‐sectionally compared the efficacy between crizotinib and savolitinib in NSCLC patients with METex14 skipping and MET amplification. Previous studies have shown that METex14 skipping exists independently as an oncogenic driver gene and is less likely to coexist with other mutations.^13^ However, in our cohort of 17 METex14 skipping patients, three patients were detected to have accompanying *EGFR* mutations which was significantly higher than the data reported in the previous literature of 0.65%, and may have been due to the bias introduced by the small sample.^14^

The PROFILE 1001 study was the first trial to formally evaluate the efficacy of crizotinib in NSCLC patients with METex14 skipping. Of the 65 patients included, the ORR was 32%, the DCR was 77.0%, with a median PFS of 7.3 months.^15^ However, in the subsequent phase II clinical study with an expanded sample size, the results were suboptimal, with a median PFS of only 3.6 months.^16^ The results of our study suggested that the efficacy of crizotinib for METex14 skipping may be inconsistent. Type Ib MET TKIs had higher selectivity compared to type 1a MET TKIs, which exhibited low specificity. Savolitinib, the first type Ib MET TKI marketed in China, demonstrated an ORR of 49.2%, a DCR of 93.4% with a median PFS of 6.8 months in its phase II clinical study.^17^ In our study, crizotinib demonstrated a PFS of 7.2 months compared with savolitinib of 10.1 months for patients with METex14 skipping, but with no statistically significant differences. In addition, crizotinib had an ORR of 37.5% with a DCR of 87.5%, while savolitinib had an ORR of 22.2% and a DCR of 88.9%, also without significant differences.

In contrast to the strong tumor driving properties of METex14 skipping, MET amplification exhibited weaker oncogenic driving force, which was closely related to gene copy number (GCN).^18^ MET amplification can act as either a primary driver gene or secondary to other gene mutations (e.g., *EGFR* mutations).^19^ Studies have shown that the incidence of primary MET amplification is approximately 1%–6%, while secondary MET amplification following EGFR TKI resistance is about 4%–31%.^7^ A subgroup analysis of the PROFILE 1001 study explored the efficacy of crizotinib for MET amplification NSCLC patients.^20^ The results showed that crizotinib demonstrated an ORR of 28.9% with a median PFS of 5.1 months for patients with MET amplification. In contrast to crizotinib, savolitinib demonstrated a superior survival benefit in NSCLC patients with MET amplification. The TATTON study explored the efficacy and safety of savolitinib in combination with osimertinib in patients with MET amplification after EGFR TKI resistance.^21^ Part B2/B3/D explored the survival benefit with savolitinib in combination with osimertinib in patients who had not been treated with triple‐generation EGFR TKIs. In these cohorts, the ORR was approximately 62%–67% and the median PFS was 9.0–11.1 months. However, it was difficult to determine whether the survival benefit was due to the role of MET TKI or triple EGFR TKI. In contrast, Part B1 explored the survival benefit of savolitinib in combination with osimertinib in patients previously treated with triple‐generation EGFR TKI.^21^ In this cohort, ORR dropped to 33% with a shortened median PFS of 5.5 months, which showed no significant difference from the data of crizotinib in the PROFILE 1001 study. However, there were many differences in the design of these two studies. The TATTON study used the regimen of savolitinib in combination with EGFR TKIs, whereas PROFILE 1001 used crizotinib alone. Therefore, they cannot be directly compared cross sectionally. Wang et al. conducted a single‐center retrospective study exploring the efficacy of crizotinib in combination with EGFR TKIs in patients with MET amplification after EGFR TKI resistance, obtaining an ORR of 81.8% with a median PFS of 5.8 months.^22^ Although its design was able to roughly match that of the TATTON study, the results may be more biased due to its small sample size (only 11 cases).

Crizotinib, classified as a type Ia MET TKI, is an ATP‐competitive multitarget inhibitor that inhibits several targets, including MET and ALK, but has a relatively weak inhibitory effect on MET. Savolitinib is a type Ib MET TKI, which can bind to ATP adenine with high specificity. Theoretically, the inhibitory effect of type Ib MET TKI is stronger than that of type Ia. In our MET amplification cohort, real‐world data confirmed this speculation. Both in combination with EGFR TKIs, savolitinib demonstrated a tendency of superior PFS benefit in patients with MET amplification than crizotinib (*p* = 0.05). The ORR of the savolitinib group was 33.3% with a median PFS of 7.1 months, similar to the TATTON Part B1 cohort. In contrast, the crizotinib group had an ORR of 27.3% with a median PFS of only 1.4 months. Notably, 54.5% of patients experienced disease progression at the time of first evaluation. This data is worse than that of the PROFILE 1001 study and the data of Wang et al., which may be due to the difference in the definition of MET amplification.

The standard detection method for MET amplification is MET/CEP7 by FISH, whereas MET gene copy number alone may not be sufficiently accurate.^23^ In the TATTON study, MET/CEP7 ≥ 2 was equated to MET GCN ≥5. In the PROFILE 1001 study, MET amplification was defined as MET/CEP7 ≥ 1.8. However, we included all patients with MET GCN ≥2 only if MET TKI was consumed, which may have resulted in more patients with low MET amplification being included. Studies have shown that high MET amplification is associated with strong oncogenic driving character.^24^ A subgroup analysis of the PROFILE 1001 study showed that the ORR in the high MET amplification group (MET/CEP7 ≥ 4.0) was 38.1% with a median PFS of 6.7 months, which was significantly higher than that of the medium or low MET amplification group.^25^ In our study, the correlation between MET amplification GCN and prognosis likewise existed. High MET amplification GCN was associated with better MET TKI efficacy. However, the accuracy of GCN to be a predictor was rather poor, with the *R* ^2^ below 0.1. It also suggested that patients with low MET amplification may possibly benefit from MET TKIs.

A meta‐analysis showed that the most common adverse reactions to crizotinib included peripheral edema, gastrointestinal reactions, liver injury, and visual impairment,^26^ while peripheral edema, gastrointestinal reactions, and liver injury were also very common to savolitinib.^27^ In our study, the incidence of adverse reactions was slightly higher in the savolitinib than the crizotinib group, especially for peripheral edema (60.0% vs. 26.3%). However, due to the retrospective nature of this study, mild adverse reactions were not recorded in detail and may have been overlooked. Thus, we only analyzed three most common adverse reactions (peripheral edema, gastrointestinal reactions and liver injury). Peripheral edema is the most important adverse reaction to MET TKIs that needs to be taken seriously. It is mainly generated by increased intravascular fluid entering the tissue interstitium or decreased fluid return through capillaries or lymphatic vessels.^28^ The etiology of MET inhibitor‐induced edema remains unclear. Kunimasa et al. suggested that it may due to the inhibition of HGF‐mediated signaling pathways in the peripheral vascular endothelium. Under physiological conditions, HGF prevented VEGF‐mediated endothelial permeability increase. MET inhibitors, which act on the HGF/MET signaling pathway, would disrupt this balance and lead to increased fluid leakage.^29^ Our study also supported the possibility of this conjecture that type 1b MET TKI (savolitinib), with higher specificity, has a higher incidence of peripheral edema than type 1a MET TKI (crizotinib).

Undeniably, there were some limitations in our study. First, the sample size of our single‐center study was small. In the future, multicenter and large‐scale studies are needed for further demonstration. Second, due to insufficient overall survival data of patients for statistical analysis, we will further extend the follow‐up period. Third, our study was retrospective in character, baseline information could not be matched exactly and thus selective bias was inevitable. The unequal market prices of savolitinib and crizotinib may cause variation in patients' choice. Although we performed matching analysis and found no statistical differences, the degree of matching was still limited. Fourth, some mild adverse reactions that were not recorded in the medical record system may have resulted in missing data.

In summary, we conducted a single‐center retrospective clinical study to explore the efficacy and safety of crizotinib and savolitinib in MET positive NSCLC patients. For METex14 skipping, the efficacy of savolitinib and crizotinib did not show significant difference. For MET amplification, savolitinib showed significantly better efficacy than crizotinib. Peripheral edema, gastrointestinal reactions and liver injury were the main adverse effects for both types of MET TKI, but the incidence of peripheral edema was higher in savolitinib than crizotinib.

## AUTHOR CONTRIBUTIONS

Kang Miao, Xiaotong Zhang: Drafting the manuscript and revising it for important intellectual content. Hanping Wang: Provision of clinical data, article revision and correction. Li Zhang, Xiaoyan Si: Substantial contributions to the conception or design of the work and final approval of the version to be published.

## FUNDING INFORMATION

No funding was received to assist with the preparation of this manuscript.

## CONFLICT OF INTEREST STATEMENT

The authors have no competing interests to declare that are relevant to the content of this article.

## Supporting information


**Table S1.** Patient detail information.

## Data Availability

The data that support the findings of this study are available from the corresponding author upon reasonable request.
